# Tb1, a Neurotoxin from *Tityus bahiensis* Scorpion Venom, Induces Epileptic Seizures by Increasing Glutamate Release

**DOI:** 10.3390/toxins12020065

**Published:** 2020-01-21

**Authors:** Emidio Beraldo Neto, Lucas Alves de Freitas, Daniel Carvalho Pimenta, Ivo Lebrun, Ana L. A. Nencioni

**Affiliations:** 1 Laboratory of Pharmacology, Butantan Institute, Av. Dr. Vital Brazil 1500, São Paulo 05503-900, Brazil; emidio.beraldo@gmail.com (E.B.N.); lcfreitas1001@gmail.com (L.A.d.F.); 2 Laboratory of Biochemistry and Biophysics, Butantan Institute, Av. Dr. Vital Brazil 1500, São Paulo 05503-900, Brazil; dcpimenta@butantan.gov.br (D.C.P.); ivo.lebrun@butantan.gov.br (I.L.); 3 Post-Graduation Program in Toxinology of Butantan Institute, Av. Dr. Vital Brazil 1500, São Paulo 05503-900, Brazil

**Keywords:** scorpion toxins, hippocampus, seizures, neurotransmitters, neuronal damage, epilepsy

## Abstract

Here, we report the neurotoxic effects aroused by the intracerebral injection (in rats) of Tb1, which is a neurotoxin isolated from *Tityus bahiensis* scorpion venom. Biochemical analyses have demonstrated that this toxin is similar to the gamma toxin from *T. serrulatus*, which is a β-scorpion toxin that acts on sodium channels, causing the activation process to occur at more hyperpolarized membrane voltages. Male Wistar rats were stereotaxically implanted with intrahippocampal electrodes and cannulas for electroencephalographic recording and the evaluation of amino acid neurotransmitters levels. Treated animals displayed behavioral and electroencephalographic alterations similar to epileptiform activities, such as myoclonus, wet dog shakes, convulsion, strong discharges, neuronal loss, and increased intracerebral levels of glutamate. Scorpion toxins are important pharmacological tools that are widely employed in ion channel dysregulation studies. The current work contributes to the understanding of channelopathies, particularly epilepsy, which may originate, among other events, from dysfunctional sodium channels, which are the main target of the Tb1 toxin.

## 1. Introduction

Sodium channels play an essential role by establishing cell excitability and maintaining the conductance of excitable cells [1]. On the other hand, several nervous system diseases may be correlated to them [1], such as epilepsy, pain, brain tumors, neural trauma, and multiple sclerosis [2]. Epilepsy is a quite common neurological syndrome [3] characterized by the occurrence of recurrent, unprovoked seizures [4] that are in part explained by an imbalance between excitatory and inhibitory conductance in the brain [5].

Despite the large availability of antiepileptic drugs (AED), there are a percentage of treatment-resistant individuals [6,7]. Moreover, AED often may exert strong side effects [8]. Therefore, there is a clear need to develop new drugs to aid such patients by improving their quality of life.

Animal venoms are rich sources of biologically active molecules that can be useful tools in the study of biological systems. Scorpion neurotoxins, particularly, have been shown to modulate sodium channel functions by modifying their permeability [9]. Such toxins have been extensively studied, and their ability to induce convulsion and neuronal loss in experimental models has already been widely demonstrated. Toxins isolated from the Brazilian scorpions *T. serrulatus* and *T. bahiensis* are able to induce seizures in rats and damage hippocampal areas when intracerebrally administered [10,11,12]. The same alterations can be observed when toxins from the Chinese scorpion *Buthus martensi* are intrahippocampally or intracerebroventricularly injected in rats [13,14]. On the other hand, the intracerebroventricular injection of a pandinustoxin-Kα toxin from *Pandinus imperator* scorpion induces limbic and clonic-tonic seizures in mice [15].

Such toxins have also been employed not only as tools for analyzing the effects of ion channel dysregulation, but also as models for evaluating mechanisms that generate neuronal injury. These studies aimed to contribute to the understanding of seizures and related mechanisms in addition to clarifying neurotransmitter-releasing processes. The development and characterization of new experimental models for the study of seizure processes will always be relevant, since such events present different etiologies.

*Tityus bahiensis* is a Brazilian scorpion that is responsible for several accidents in the southeast region [16,17,18]. Their specific biological requirements, such as the habitat temperature and humidity variation, and their sexual reproduction restrict them to this geographic region [19]. This adaptation to a food-competitive environment may have resulted in a selective process, ultimately culminating in the specific animal’s venom composition and biological development. 

Despite no more than 10 toxins having already been isolated from this venom [20,21,22,23], their mechanisms of action remain poorly explored [12,24,25].

In this study, we have examined the ability of Tb1 to induce seizures and epileptiform behavior in rats as well as the release of endogenous glutamate from hippocampal nerve terminals. The current study provides information regarding the toxin’s mechanism of neuronal action in the central nervous system, which would be useful for understanding the involvement of sodium channels in epileptogenesis. The hippocampus was selected for the toxin administration because, being a component of the limbic system, it is much more sensitive to seizures than other intracranial regions [26,27]. Moreover, this is the model adopted by our laboratory for testing the central effects of toxins. Finally, the dose employed in this study is based on our previous experience when studying the central effects of other scorpion toxins [12].

## 2. Results

### 2.1. Biochemical Characterization of Tb1

Tb1 was obtained by C18-RP-HPLC, according to Beraldo-Neto [12], from Fraction II of the Sephadex G25 (fine) size exclusion chromatography of *T. bahiensis* venom, which was previously termed TbII-II. Such fraction was further processed as follows.

**Figure 1:**
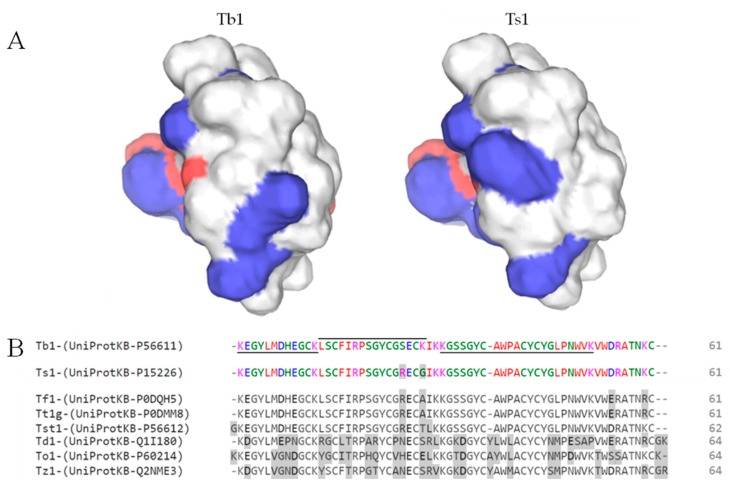
(**A**) 3D model of Tb1 peptide created with the SWISS-MODEL [29] based on the Ts1 structure proposed by Pinheiro [30], highlighting the different charges of each peptide in blue and red, respectively positive and negative. (**B**) Sequence alignment analysis with Clustal O (1.2.4) of gamma toxins of the main *Tityus* species with emphasis on different amino acids regarding Tb1. After Tb1 (*Tityus bahiensis*), Ts1 (*Tityus serrulatus*), Tf1 (*Tityus fasciolatus*), Tt1g (*Tityus trivittatus*), Tst1 (*Tityus stigmurus*), Td1 (*Tityus discrepans*), To1 (*Tityus obscurus*), and Tz1 (*Tityus zuliani*) sequence alignment analysis, Tb1 was subjected to trypsin digestion solution, and the peptidomic analysis was performed against a transcriptome database obtained from the venom gland of *Tityus bahiensis*. The identified peptides for Tb1 are highlighted.

A trypsin- digested proteomic approach was performed, and the results were analyzed against a transcriptome database obtained from the venom gland of *T. bahiensis*. It was possible to identify the presence of a major toxin Tb1 (UniProt code: P56611); tryptic peptides are highlighted in Figure 1B, and Figure S1 shows all the peptides found in the analysis. The complementary Table S1 further clarifies the sequences and logs for result reliability, along with Figures S2–S15, which show the spectra with the identified ion tables and the error map. Figure 1B also presents the alignment of other gamma toxins from the major *Tityus* species.

Since Tb1 and Ts1 (from *Tityus serrulatus*) venom are highly similar (96.72% identity, two different amino acids), a structural comparative analysis (Figure 1A) was performed by comparing the structure of Ts1, which was firstly isolated and sequenced by Possani [28], and a SwissModel generated 3D structure of Tb1 (Figure 1A). Although minor, the two-amino acid substitution in Tb1 (Ser for Arg and Lys for Gly) is likely to provoke profound changes in the peptide surface charge distribution, as presented in Figure 1A. 

**Figure 2:**
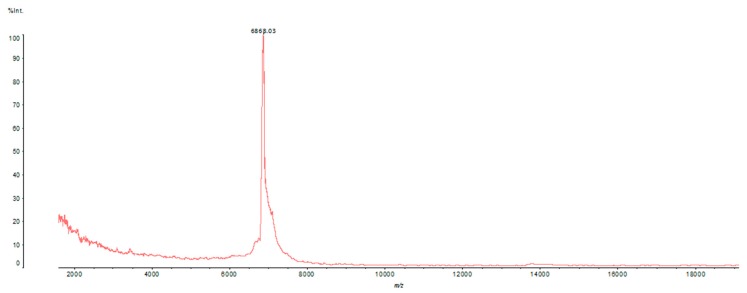
Matrix-assisted laser desorption ionization-time of flight (MALDI-TOF)/MS profile of Tb1. It is possible to observe the presence of a single peptide (6868 Da).

In order to evaluate the molecular mass and fractionation efficiency for Tb1, matrix-assisted laser desorption ionization-time of flight (MALDI-TOF) analysis was performed. A single component with molecular mass of 6868.03 Da was detected (Figure 2).

### 2.2. Behavioral, Electrographic, and Histopathological Effects of Intrahippocampal Injection of Tb1

**Figure 3:**
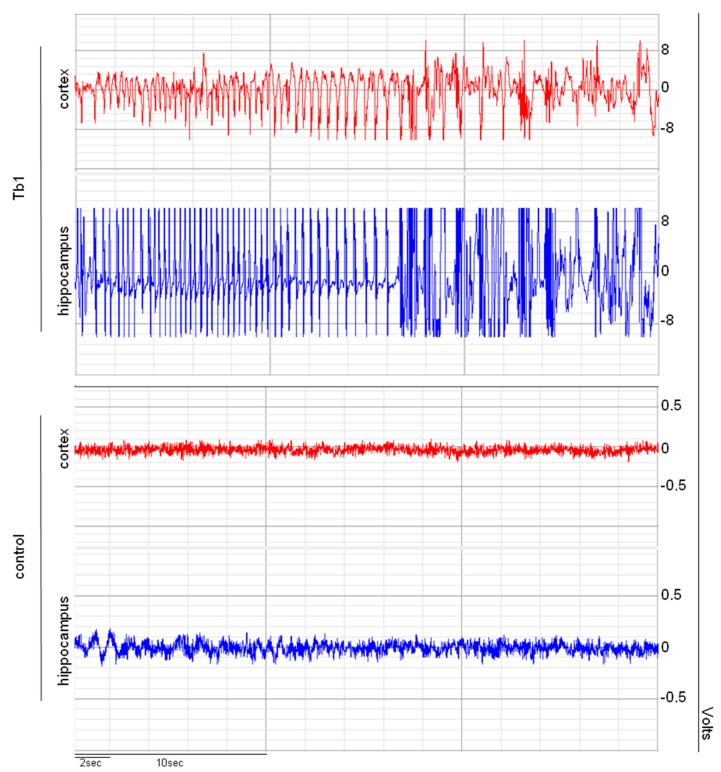
Representative electrographic recording pattern in the hippocampus and cortex induced by the intrahippocampal injection of Ringer’s solution (1.0 µL, control group) or Tb1 (2.0 µg/µL). Continuous recording were performed for a period of 4 h.

**Figure 4:**
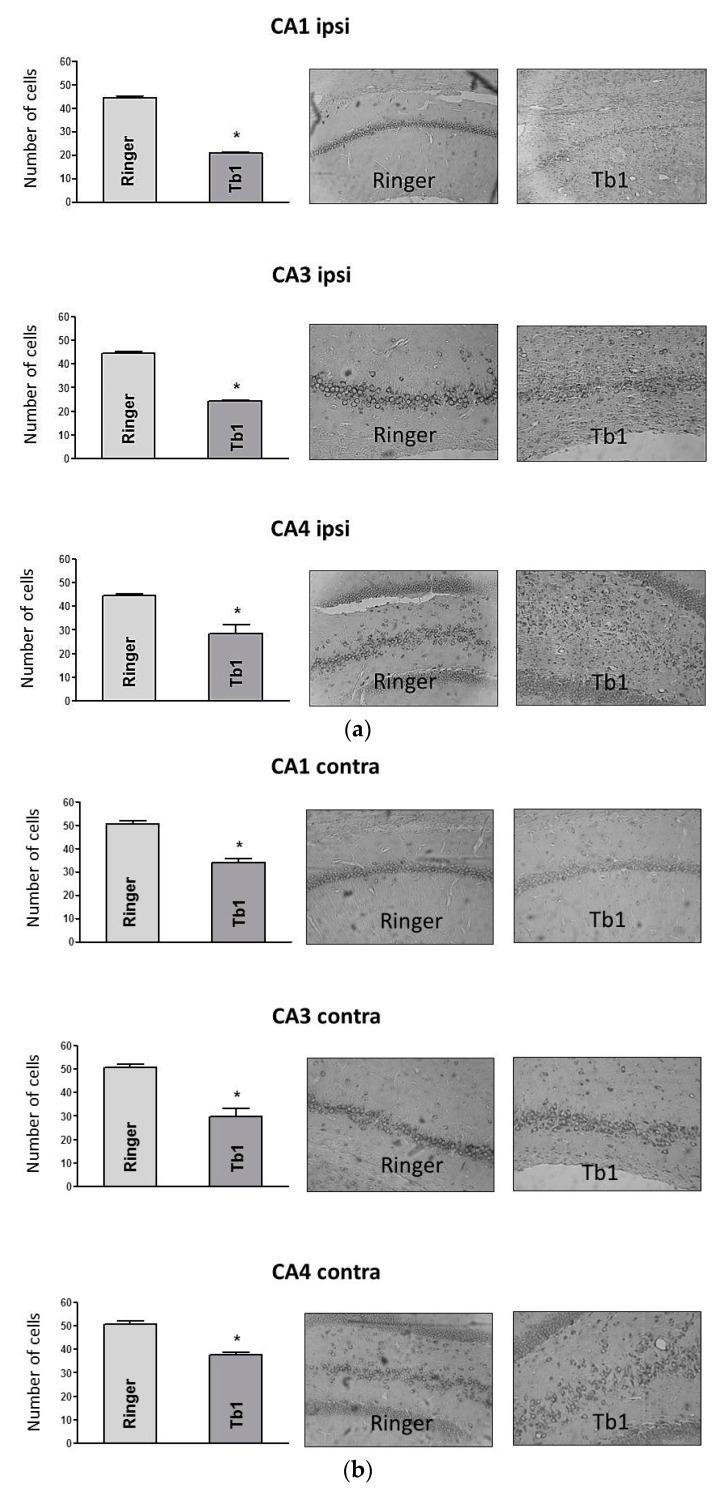
Histological sections of rat brains (10 µm) analyzed by light microscopy at 400×. The analysis refers to (**a**) the layer of injected (ipsi) side with Tb1 (2.0 µg/µL, *n* = 6) or Ringer’s solution (1.0 µL, *n* = 6) and (**b**) the non-injected (contra) side of the hippocampal CA1, CA3, and CA4 areas. Data are expressed as the mean± SD. * *p* < 0.05 compared to the control group (ANOVA followed by Tukey’s test).

The intrahippocampal injection of Tb1 elicited strong respiratory difficulty in four out of six of the animals, and five out of six had myoclonus and wet dog shakes (WDS) (Table 1). The parameters prostration and convulsion were also altered in some animals (2/6 and 3/6 respectively) but not in a significant manner (Table 1). Most animals also presented spikes and intense epileptiform discharges (Table 1 and Figure 3), and there was a reduction in the number of intact neurons in the CA1, CA3, and CA4 ipsilateral and contralateral areas (Figure 4). The animals from the control group (Ringer) showed no behavioral (Table 1), electrographic (Table 1), or histopathological alterations (Figure 4).

### 2.3. Effects of Intrahippocampal Injection of Tb1 on the Extracellular Level of Amino Acid Neurotransmitters in Rats

**Figure 5:**
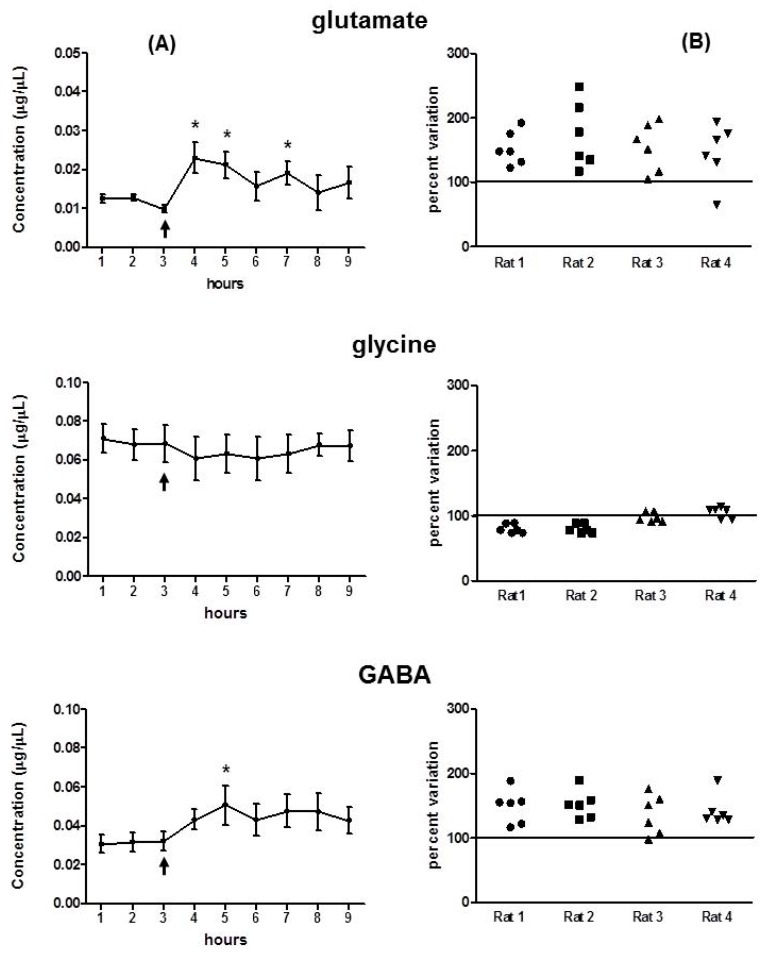
Extracellular levels of glutamate, glycine, and GABA as determined by microdialysis in the CA1 area of the hippocampus in conscious rats (*n* = 4) before (up to 3 h) and after injection of Tb1 (2.0 µg/µL) in the same area. (**A**) Average values from all animals per each time point. The arrow denotes the injection time. Data are expressed as mean ± SD. * *p* < 0.05 compared to the control group (ANOVA followed by Tukey’s test). (**B**) The set of points means the extracellular levels of glutamate, glycine, and GABA reached by each rat after injection of the toxin (from 3 to 9 h). Horizontal bars indicate the mean baseline level of the neurotransmitter before injection (mean of samples 1, 2, and 3).

Tb1 increased glutamate levels right after the injection, which remained elevated for the following 4 h. After that, the levels slowly tended to diminish, still remaining above baseline; however, this was not performed in a statistically significant manner (Figure 5). Interestingly, this effect could not be observed for glycine, which maintained its mean values throughout the experiment (Figure 5A). The level of GABA tended to rise after injection, although this was not in a statistically significant manner, except for the 5-h point. Figure 5B shows that glutamate and GABA levels remain above the baseline at all points analyzed for all animals. 

## 3. Discussion

Although of medical importance in Brazil, not many reports describing the *T. bahiensis* venom and/or toxins are available, and the scarce literature is limited to describing the purification and characterization of few venom components [12,22,31,32]. Few studies describe the central effects of isolated molecules (or fractions), showing for example that when intravenously injected in rats, they are able to induce spontaneous convulsion, and when injected in the hippocampus, seizures and behavioral alterations are observed [24]. Electrographic and behavioral alterations increase in the extracellular levels of glutamate, and neuronal loss was observed after intrahippocampal injection of the toxin Tb V-4, besides an increase in the cytosolic calcium concentration [25]. Recently, it was demonstrated that the intrahippocampal injection of Tb II-I in rats induces seizures and neuronal loss as well as increase in the hippocampal level of TNF-α and IL-6 [12].

The biochemical analyses performed in this study demonstrated that the currently described toxin was, in fact, previously described by Becerril [32]. Therefore, we adopted the same nomenclature, Tb1. It is a β-type scorpion toxin active at site 4 of the voltage-gated sodium channel, causing an amplitude reduction in current and a voltage-dependence activation shift to more hyperpolarized potentials [33]. It is homologous to the gamma toxin (also known as Ts1, TsTX-I, Ts VII, or toxin γ) isolated from *Tityus serrulatus* venom, which is one of the most studied and well characterized toxins, being the major and most potent component of the venom [34]. Several homologous toxins (Figure 1B) have been identified in other *Tityus* species such as Tst1 from *T. stigmurus* [32], Tz1 from *T. zulianus* [35], TdI from *T. discrepans* [36], Tt1g from *T. trivittatus* [37], and Tf1a from *T. fasciolatus* [38].

Although classified as a β-toxin, with classic action on the Nav1.2, Nav1.3, Nav1.4, and Nav1.6 channels, Ts1 has its own action repertoire; in DmNaV1, its effect resembles toxins that inhibit or slow down the rapid inactivity of sodium channels after binding to site 3, with increasing current amplitude and the appearance of persistent currents [39]. Taking into account that surface Ts1 residues (12Lys, 39Trp, and 54Trp) are crucial for the functional interaction with channels [40], we built a 3D Tb1 model and compared it with the Ts1 3D structure (Figure 1A).

Although only two amino acids differ between these two toxins, these substitutions are critical; both involve charged amino acids. That is, 25S-> R and 28K-> G (Figure 1B). In addition, these amino acids are exposed in Tb1; therefore, they are likely to participate in the receptor binding process, and specifically, 28K is on the face that interacts with the sodium channel. Thus, it can affect its biological effects.

Some of these toxins have been investigated regarding the systemic effects and the interaction on sodium channels [34,35,36,37,38]. On the other hand, the central effects were evaluated only with Ts1 [41,42,43] and Tb1 in the current study.

Ts1 has been shown to evoke glutamate release from rat cortical synaptosomes in a dose-dependent manner and to increase sodium and calcium concentrations [41]. When intracerebrally injected in rats, it causes epileptiform discharges, paralysis in hind limbs, and respiratory distress, followed by death at higher doses [42]. Moreover, Ts1 alters cytokine levels without damaging neurons or altering the hippocampal concentration of glutamate [43]. Similarly, the intracerebral injection of Tb1 in rats causes epileptic-like discharges, but apparently, it is more toxic, since it caused an intense neuronal damage and increase in the level of hippocampal glutamate. However, the dose used in this study (2.0 µg, the group’s standard when performing pharmacological characterization) was higher than those used by Teixeira [42] and Rodriguez [43] (0.005 and 0.125 µg, respectively). Such a difference could explain the more intense observed effects.

Neuronal loss may actually be a consequence of increased glutamate levels in a process known as excitotoxicity, which is characterized by the binding of excess glutamate to its receptors, promoting an abnormal calcium influx in the cells. The consequence would be the nNOS upregulation, the dysfunction of mitochondria, reactive oxygen species (ROS) production, lysosomal enzymes releasing, and ultimately, neuronal death [44]. Moreover, augmented glutamate, the main excitatory neurotransmitter in the brain, is strongly related to seizures [45,46,47]. The imbalance between excitatory and inhibitory conductance, which is mediated by glutamate and GABA respectively, triggers the convulsive process [5]. Glutamate seems to be involved also in myoclonus [48], which is considered one of many symptoms of epileptic disorder and characterized by whole-body twitch, and WDS, which is a characteristic behavior of focal limbic seizures consisting of repeated shakes of the head and the trunk [49]. All these events were observed in the test subjects, besides increased GABA levels, which may be a compensatory mechanism trying to inhibit the epileptic seizure, in an attempt to limit the progression and propagation of the stimulus responsible for the seizure [50]. The increase in GABA concentration may be a physiological response of the central nervous system trying to maintain the balance between excitatory and inhibitory activity and trying to prevent the generation of new epileptic stimuli. 

All of these events are consistent to what has already been described for other scorpion toxins [11,25,51,52], and they are in accordance with the ability of scorpion toxins to act on ion channels, modifying their functioning. Similar convulsive effects are found in clinical cases of severe envenomation worldwide [53,54,55,56,57,58].

On the other hand, the toxins’ ability to specifically interact with cellular targets makes them useful pharmacological tools for the study of ion channels and associated channelopathies. 

Epilepsy is considered a channelopathy once there is a disorder in the neuronal excitability caused by the improper functioning of sodium channels [2,59], which is frequently due to genetic mutations, mainly of the Nav1.1 subtype channel [60].

The classic antiepileptic drugs act through three basic mechanisms: the modulation of voltage-dependent ion channels, decreasing the excitatory transmission, or increasing the GABA-mediated inhibitory neurotransmission [61]. Currently, the most common drugs used in the treatment of epilepsy are sodium channel blockers [62]. However, there are types of epilepsy that are resistant to traditional antiepileptic drugs [6], making it extremely necessary to discover alternative substances. In this sense, animal toxins that modulate ion channels could be extremely useful for developing better and safer drugs. Peptides isolated from several venomous animals such as cone snails, spiders, wasps, and scorpions have been considered potential antiepileptic agents [8]. In this context, Tb1 might be a valuable tool in the study of the involvement of sodium channels in seizures, helping to understand the participation of these channels in the clinical manifestations aroused by abnormal cell excitability.

## 4. Conclusions

Tb1 acts on sodium channels, promoting abnormal functioning that results in an excessive release of glutamate. Overloaded glutamate causes behavioral and electrographic epileptiform alterations and intense neuronal injury. Due to the need for the discovery of novel active molecules that could aid in understanding and treating channelopathies, this toxin can be an important tool to help this kind of study.

## 5. Materials and Methods 

### 5.1. Purification and Biochemical Characterization of Tb1

The lyophilized scorpion venom was provided by the Strategic Nucleus of Venoms and Antivenoms of Butantan Institute, São Paulo, Brazil.

The isolated toxin was obtained by the same methodology used by Beraldo-Neto [12] in the high-performance liquid chromatography (HPLC) of Fraction II derived from the size-exclusion chromatography of *T. bahiensis* venom, which was collected as peak 2 or as identified in the work as Tb1.

Tb1 was analyzed using a matrix-assisted laser desorption ionization-time of flight (MALDI-TOF) mass spectrometer (Axima Performance, Shimadzu, Kyoto, Japan). First, 1 µl of Tb1 fraction was co-crystallized with 1 µl of sinapinic acid matrix (saturated solution prepared in 50% ACN/0.1% acetic acid) in the plate and dried at room temperature. The mass spectrum was obtained in the 50–20,000 mass/charge (m/z) range, in linear positive mode with laser power at 120.

Then, an aliquot was subjected to in-solution digestion for proteomic analysis. The selected peak was adjusted to pH (>7.0) with 5 µl of a buffer solution (bicarbonate of ammonia 50 mM), after which 5 µl of dithiothreitol (100 mM) was added to the sample and heated at 60 °C for 30 min. Then, 2.5 µl of iodoacetamide (200 mM) was added and kept at room temperature and protected from light for at least 30 min. Afterwards, 10 μL of trypsin (40 ng/μL) was added to the solution, and the incubation was performed overnight at room temperature. The reaction was stopped adding 50% ACN/5% acetic acid. All the reagents were purchased from Sigma-Aldrich (St. Louis, MO, USA).

The sample was analyzed by liquid chromatography-mass spectrometry using an ESI-IT-TOF system coupled to binary an ultra-fast liquid chromatography system (UFLC) (20A Prominence, Shimadzu). Each sample was loaded in a C18 column (C18, 50 µm; 50 × 2.1 mm) in a binary solvent system: (A) water to acetic acid (999:1, *v:v*) and (B) ACN to water to acetic acid (900:99:1, *v:v:v*). The column was eluted at a constant flow rate of 0.2 mL/min with a 0–40% gradient of Solvent B over 35 min. The eluates were monitored by a Shimadzu SPD-M20A PDA detector before introduction into the mass spectrometer. The interface voltage was 4.5 KV; the capillary voltage was 1.85 KV at 200 °C; and the fragmentation was induced by argon collision, at 55% ‘energy’. MS spectra were acquired under positive mode and collected in the 350–1400 mass/charge (m/z) range. MS/MS spectra were collected in the 50–1950 m/z range. LCD Shimadzu raw data were converted (LCMS Protein Postrun, Shimadzu, Kyoto, Japan) to Mascot Generic Format (MGF) files prior to analyses. Peaks Studio V7.0 (BSI, Toronto, Canada) was used for data processing (de novo peptide sequencing and proteomic identification) [63]. Proteomic identification was performed according to the following parameters: error mass (MS and MS/MS) set to 0.2 Da; methionine oxidation and carbamidomethylation as variable and fixed modification, respectively; trypsin, as the cleavage method; maximum missed cleavages (3), maximum variable post-translational modifications (PTMs) per peptide (3), and non-specific cleavage (1). The sample was analyzed against a transcriptome database (9151 search entry) obtained from the venom gland of *T. bahiensis* (GenBank: GBXR00000000.1) 

### 5.2. Animals and Surgical Procedures

Experiments were performed on 16 male Wistar rats with body mass 240–260 g. The animals were maintained in air-conditioned rooms, with temperature 22 °C and a 12/12 h light–day cycle. The study was approved by the Ethics Committee on Animal Use of the Butantan Institute (CEUAIB) in the meeting of 26 July 2018 (permission number 9998050718).

The animals were anesthetized with a mixture of 10% ketamine and 2% xylazine (1.3 mg/kg, i.p.) and fixed in a stereotaxic apparatus. After local asepsis, the skull was exposed, and cannulas and/or electrodes were intrahippocampally implanted according to the Stereotactic Atlas of Paxinos and Watson [64]. The system was fixed to the skull through stainless steel screws and dentary cement. Then, the skin was sutured and the cannulas and/or electrodes were exposed. After a recovery period (48 h) in individual metal cages with free access to water and food, the animals were intrahippocampally injected with Tb1 (2.0 µg/µL) or Ringer solution (1.0 µL). The injection was performed slowly (0.25 μL/min) through a system consisting of a 30 G needle inserted into the guide cannula and connected through a polyethylene catheter to a 5 μL Hamilton microsyringe. Immediately after the injection, the animals were re-placed in their experiment cages.

### 5.3. Electrographic and Behavioral Observation

The animals were individually placed in acrylic boxes (30 × 20 × 30 cm) in a Faraday cage, freely moving for 30 min for habituation. Afterwards, the animals were connected to an electrical activity recorder (BIOPAC System Inc., Goleta, CA, USA, Model MP150) and were analyzed according to their behavior and electrographic activity during an initial period of 30 min (basal activity). Then, they were intrahippocampally injected (experimental group with Tb1, n = 6, and control group with Ringer solution n = 6) and continuously observed over 4 h. Isolated or clustered spikes and moderate and/or intense discharges were considered epileptiform activity. Respiratory difficulty, myoclonus, wet dog shakes (WDS), and salivation were considered behavioral alterations. 

Statistical analyses were performed by Fisher’s test, and the level of significance was set at *p* < 0.05.

### 5.4. Glutamate, Glycine, and GABA Level Assessment

A group of four animals were placed in individual acrylic cages where they remained for about 30 min for habituation. Then, microdialysis probes (CMA/11 microdialysis probes, Stockholm, Sweden; membrane length 4 mm) were connected to the guide cannulas, and sample collection was started. After a 45–50-min equilibration period, perfusates were collected every 60 min. After the collection of the first three samples (whose average was used to establish the baseline values of glutamate, glycine, and GABA, which were defined as 100%), the animals received an intrahippocampal injection of Tb1 (2.0 μg/µL) and an additional six samples were collected, making a total of nine samples per animal.

Immediately after the perfusate collection, the samples were dried in SpeeVac (B446—Savant, USA) and kept at −80 °C until the analysis. Perfusates were analyzed for amino acid content using high-performance liquid chromatography (UFLC - Shimadzu) along with fluorometric detection after precolumn derivatization with phenyl-isothiocyanate. The substances were recognized according to their retention time in the chromatographic column, comparing them to an amino acid standard of known concentration, as described previously by Heinrikson and Meredith [65].

Statistical analyses were performed by ANOVA followed by Tukey’s test, and the level of significance was set at *p* < 0.05.

### 5.5. Histological Analysis

One week after the electrographic observations or microdialysis collection, the animals were deeply anesthetized with carbon dioxide (CO_2_) and perfused by cardiac puncture with phosphate-buffered saline (PBS) and 10% buffered formalin. The brains were removed, processed, and embedded in Paraplast^®^ (manufactured by Oxford Labware, St. Louis, MO, USA) for histopathological analysis. The tissues were sliced (10 μm) in a microtome (Leica Biosystems Nussloch GmbH, Wetzlar, Germany, model RM 2235). The slices were stained with cresyl violet and evaluated by light microscopy. Intact pyramidal neurons with a visible nucleus and nucleolus were counted in the CA1, CA3, and CA4 hippocampal areas, ipsilateral and contralateral to the injection site. 

Statistical analyses were performed by ANOVA followed by Tukey’s test, and the level of significance was set at *p* < 0.05.

## Figures and Tables

**Table 1:** Behavioral and electrographic effects observed after intrahippocampal injection of Tb1.

Observed Effects	Parameters	Tb1 (2.0 μg/μL) *n* = 6	Ringer (1 μL) *n* = 6
Behavioral effects	Prostration	2/6	0
	Respiratory difficulty	4/6 *	0
	Myoclonus	5/6 *	0
	WDS	5/6 *	0
	Convulsion	3/6	0
Electrographic effects	Spikes	5/6 *	0
	Discharges	5/6 *	0

The statistical analysis was performed using Fisher’s exact test, * *p* < 0.05.

## Supplementary Materials

The following are available online at https://www.mdpi.com/2072-6651/12/2/65/s1, Figure S1: Result of the coverage and reliability of identification of Tb1 against Arachnida database and the respective peptides found, Table S1: List of peptides identified for Tb1, Figure S2: Spectrum from ion 1919.8328 tryptic peptides identified for Tb1 with ion table and error map, Figure S3: Spectrum from ion 2043.8794. tryptic peptides identified for Tb1 with ion table and error map, Figure S4: Spectrum from ion 1828.7007 tryptic peptides identified for Tb1 with ion table and error map, Figure S5: Spectrum from ion 1505.6067 tryptic peptides identified for Tb1 with ion table and error map, Figure S6: Spectrum from ion 1465.6329 tryptic peptides identified for Tb1 with ion table and error map, Figure S7: Spectrum from ion 1458.6425 tryptic peptides identified for Tb1 with ion table and error map, Figure S8: Spectrum from ion 1412.6177 tryptic peptides identified for Tb1 with ion table and error map, Figure S9: Spectrum from ion 1337.538 tryptic peptides identified for Tb1 with ion table and error map, Figure S10: Spectrum from ion 1198.5804 tryptic peptides identified for Tb1 with ion table and error map, Figure S11: Spectrum from ion 1143.4325 tryptic peptides identified for Tb1 with ion table and error map, Figure S12: Spectrum from ion 1135.5485 tryptic peptides identified for Tb1 with ion table and error map., Figure S13: Spectrum from ion 1559.686 tryptic peptides identified for Tb1 with ion table and error map, Figure S14: Spectrum from ion 812.4545 tryptic peptides identified for Tb1 with ion table and error map, Figure S15: Spectrum from ion 794.4109 tryptic peptides identified for Tb1 with ion table and error map.
