# Supplementary material for: Tb1, a Neurotoxin from Tityus bahiensis Scorpion Venom, Induces Epileptic Seizures by Increasing Glutamate Release
Source: Toxins (Basel). 2020 Jan 21;12(2):65. doi: 10.3390/toxins12020065 (PMC7076872; doi:10.3390/toxins12020065)
Supplement: Supplementary file 1 [file toxins-12-00065-s001.pdf]

Tb1, a neurotoxin from *Tityus bahiensis* scorpion venom, induces epileptic seizures by increasing glutamate release

Emidio Beraldo Neto Lucas Alves de Freitas, Daniel Carvalho Pimenta, Ivo Lebrun and Ana L. A. Nencioni

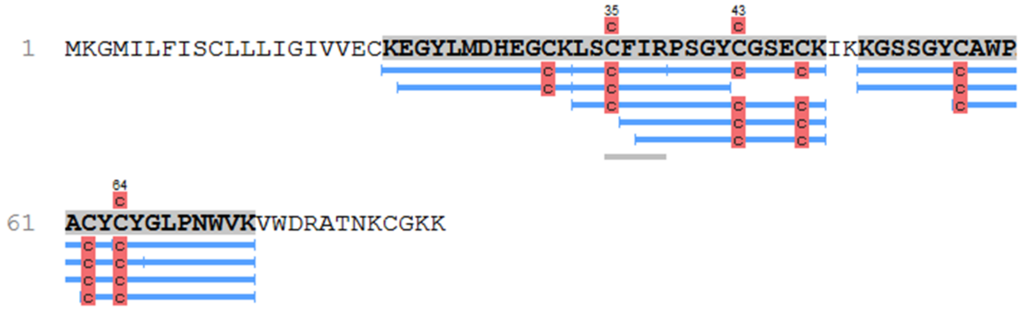

Acession	Coverage	-10lgP	Peptides	Unique Peptides	Avg. Mass
P5661	60%	176.24	14	13	9384

Figure S1. Result of the coverage and reliability of identification of Tb1 against Arachnida database and the respective peptides found.

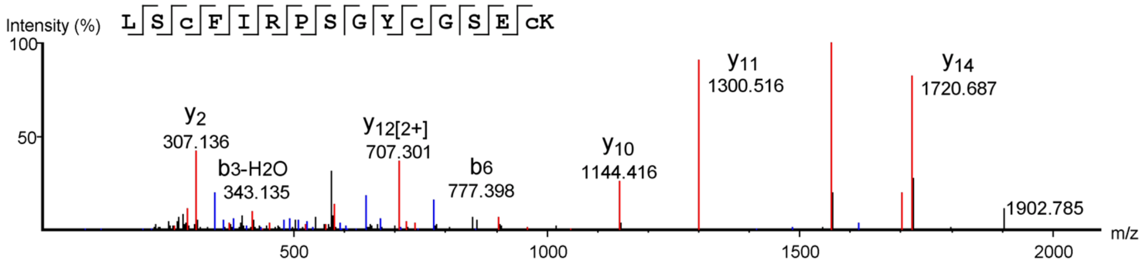

#	Immonium	b	b-H2O	b-NH3	a	c-H	b (2+)	Seq	y	y-H2O	y-NH3	y (2+)	#
1	86.097	114.092	96.081	97.065	86.145	130.111	57.546	L					16
2	60.045	201.124	183.113	184.097	173.129	217.084	101.062	S	1807.756	1789.745	1790.729	904.316	15
3	133.043	361.141	343.135	344.128	333.160	377.174	181.077	C(+57.02)	1720.687	1702.679	1703.697	860.862	14
4	120.081	508.208	490.197	491.206	480.210	524.242	254.611	F	1560.656	1542.683	1543.666	780.847	13
5	86.145	621.307	603.256	604.332	593.312	637.326	311.154	I	1413.625	1395.614	1396.598	707.301	12
6	129.114	777.398	759.398	760.381	749.413	793.427	389.204	R	1300.516	1282.530	1283.514	650.770	11
7	70.066	874.461	856.450	857.342	846.466	890.480	437.730	P	1144.416	1126.429	1127.413	572.720	10
8	60.045	961.493	943.482	944.466	933.498	977.512	481.246	S	1047.335	1029.376	1030.360	524.193	9
9	30.034	1018.514	1000.504	1001.487	990.519	1034.534	509.757	G	960.384	942.344	943.328	480.677	8
10	136.076	1181.578	1163.567	1164.551	1153.583	1197.597	591.259	Y	903.333	885.323	886.307	452.182	7
11	133.043	1341.608	1323.598	1324.581	1313.613	1357.628	671.269	C(+57.02)	740.195	722.185	723.234	370.630	6
12	30.034	1398.630	1380.619	1381.603	1370.635	1414.638	699.815	G	580.229	562.207	563.213	290.620	5
13	60.045	1485.586	1467.651	1468.635	1457.667	1501.681	743.380	S	523.210	505.207	506.191	262.098	4
14	102.055	1614.713	1596.694	1597.677	1586.709	1630.724	807.852	E	436.185	418.182	419.159	218.593	3
15	133.043	1774.735	1756.725	1757.708	1746.740	1790.754	887.868	C(+57.02)	307.136	289.126	290.104	154.072	2
16	101.108							K	147.113	129.102	130.086	74.056	1

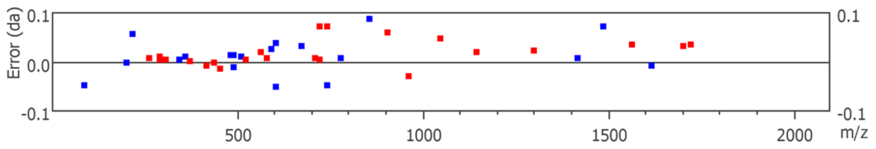

Figure S2. Spectrum from ion 1919.8328 tryptic peptides identified for Tb1 with ion table and error map. Spectrum from ion 1919.8328 tryptic peptides identified for Tb1 with ion table and error map.

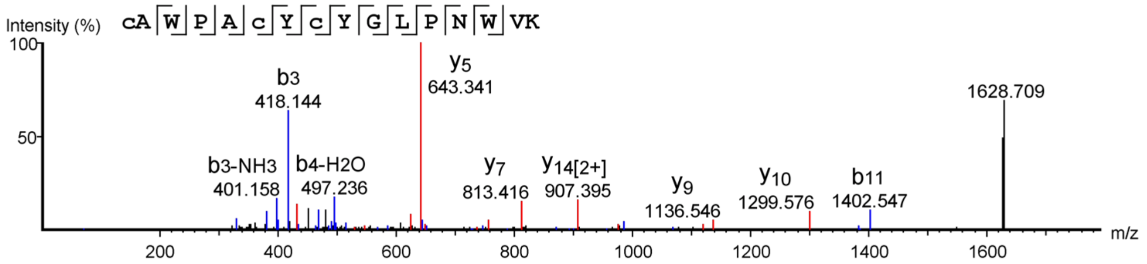

#	Immonium	b	b-H2O	b-NH3	a	c-H	b (2+)	Seq	y	y-H2O	y-NH3	y (2+)	#
1	133.043	161.038	143.028	144.011	133.043	177.058	81.019	C(+57.02)					16
2	44.050	232.076	214.065	215.049	204.081	248.095	116.538	A	1884.856	1866.845	1867.829	942.928	15
3	159.092	418.144	400.144	401.158	390.160	434.220	209.577	W	1813.819	1795.808	1796.792	907.395	14
4	70.066	515.238	497.236	498.215	487.161	531.227	258.104	P	1627.740	1609.729	1610.713	814.370	13
5	44.050	586.210	568.230	569.218	558.250	602.264	293.622	A	1530.687	1512.676	1513.660	765.843	12
6	133.043	746.248	728.256	729.252	718.280	762.295	373.638	C(+57.02)	1459.650	1441.639	1442.623	730.325	11
7	136.076	909.339	891.328	892.214	881.344	925.358	455.169	Y	1299.576	1281.609	1282.592	650.320	10
8	133.043	1069.331	1051.359	1052.342	1041.374	1085.389	535.185	C(+57.02)	1136.546	1118.545	1119.528	568.778	9
9	136.076	1232.433	1214.422	1215.406	1204.438	1248.452	616.716	Y	976.477	958.514	959.498	488.763	8
10	30.034	1289.454	1271.444	1272.427	1261.459	1305.473	645.288	G	813.416	795.451	796.435	407.231	7
11	86.097	1402.547	1384.454	1385.457	1374.543	1418.557	701.769	L	756.413	738.331	739.413	378.720	6
12	70.066	1499.591	1481.580	1482.564	1471.596	1515.610	750.295	P	643.341	625.296	626.290	322.178	5
13	87.056	1613.634	1595.623	1596.607	1585.639	1629.653	807.317	N	546.291	528.293	529.290	273.652	4
14	159.092	1799.713	1781.703	1782.686	1771.718	1815.732	900.266	W	432.250	414.250	415.234	216.630	3
15	72.069	1898.782	1880.771	1881.755	1870.787	1914.801	949.891	V	246.181	228.171	229.154	123.591	2
16	101.108							K	147.113	129.102	130.086	74.056	1

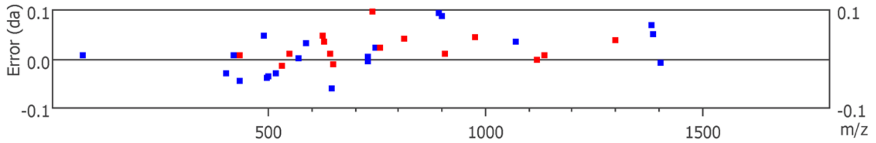

Figure S3. Spectrum from ion 2043.8794 tryptic peptides identified for Tb1 with ion table and error map.

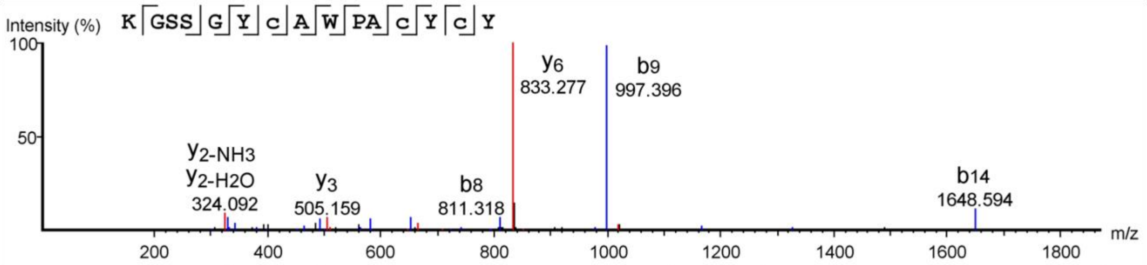

#	Immonium	b	b-H2O	b-NH3	a	c-H	b (2+)	Seq	y	y-H2O	y-NH3	y (2+)	#
1	101.108	129.103	111.092	112.076	101.108	145.122	65.051	K					15
2	30.034	186.124	168.114	169.097	158.129	202.143	93.562	G	1701.613	1683.603	1684.586	851.254	14
3	60.045	273.156	255.146	256.129	245.161	289.175	137.078	S	1644.592	1626.581	1627.565	822.796	13
4	60.045	360.188	342.112	343.161	332.126	376.207	180.594	S	1557.560	1539.549	1540.533	779.280	12
5	30.034	417.210	399.142	400.183	389.215	433.229	209.105	G	1470.527	1452.517	1453.500	735.764	11
6	136.076	580.256	562.240	563.192	552.278	596.292	290.637	Y	1413.506	1395.496	1396.479	707.234	10
7	133.043	740.287	722.293	723.312	712.309	756.323	370.652	C(+57.02)	1250.443	1232.432	1233.416	625.721	9
8	44.050	811.318	793.315	794.263	783.346	827.360	406.170	A	1090.412	1072.402	1073.385	545.706	8
9	159.092	997.396	979.337	980.393	969.425	1013.439	499.222	W	1019.386	1001.364	1002.348	510.194	7
10	70.066	1094.473	1076.462	1077.446	1066.478	1110.492	547.736	P	833.277	815.285	816.269	417.148	6
11	44.050	1165.516	1147.500	1148.483	1137.515	1181.529	583.255	A	736.243	718.232	719.216	368.621	5
12	133.043	1325.529	1307.530	1308.514	1297.546	1341.560	663.270	C(+57.02)	665.192	647.176	648.179	333.103	4
13	136.076	1488.604	1470.594	1471.577	1460.609	1504.623	744.802	Y	505.159	487.165	488.148	253.088	3
14	133.043	1648.594	1630.624	1631.608	1620.640	1664.654	824.817	C(+57.02)	342.112	324.092	325.087	171.556	2
15	136.076							Y	182.081	164.071	165.054	91.541	1

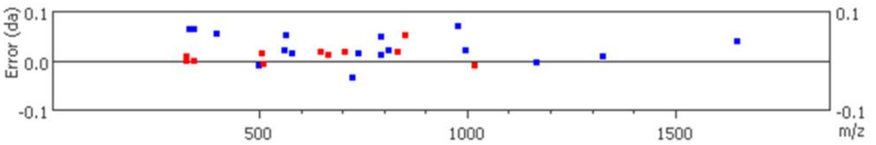

Figure S4. Spectrum from ion 1828.7007 tryptic peptides identified for Tb1 with ion table and error map.

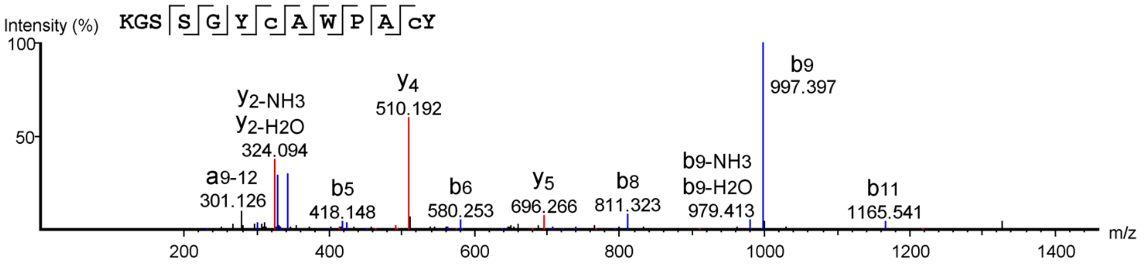

#	Immonium	b	b-H2O	b-NH3	a	c-H	b (2+)	Seq	y	y-H2O	y-NH3	y (2+)	#
1	101.108	129.103	111.092	112.076	101.108	145.122	65.051	K					13
2	30.034	186.124	168.114	169.097	158.129	202.143	93.562	G	1378.519	1360.509	1361.492	689.760	12
3	60.045	273.156	255.146	256.129	245.161	289.175	137.078	S	1321.498	1303.487	1304.471	661.249	11
4	60.045	360.188	342.104	343.112	332.128	376.207	180.594	S	1234.466	1216.455	1217.496	617.733	10
5	30.034	417.169	399.192	400.183	389.215	433.229	209.105	G	1147.434	1129.423	1130.407	574.231	9
6	136.076	580.253	562.240	563.246	552.278	596.292	290.637	Y	1090.412	1072.402	1073.385	545.706	8
7	133.043	740.279	722.250	723.277	712.309	756.323	370.652	C(+57.02)	927.349	909.336	910.339	464.171	7
8	44.050	811.323	793.340	794.314	783.346	827.360	406.170	A	767.375	749.308	750.291	384.159	6
9	159.092	997.397	979.413	980.416	969.425	1013.439	499.210	W	696.266	678.270	679.261	348.641	5
10	70.066	1094.473	1076.462	1077.446	1066.478	1110.492	547.736	P	510.192	492.199	493.198	255.601	4
11	44.050	1165.541	1147.500	1148.483	1137.515	1181.529	583.242	A	413.147	395.138	396.122	207.074	3
12	133.043	1325.541	1307.530	1308.514	1297.546	1341.560	663.270	C(+57.02)	342.104	324.094	325.089	171.556	2
13	136.076							Y	182.081	164.071	165.054	91.541	1

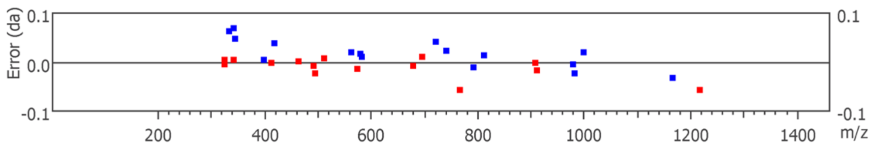

Figure S5. Spectrum from ion 1505.6067 tryptic peptides identified for Tb1 with ion table and error map.

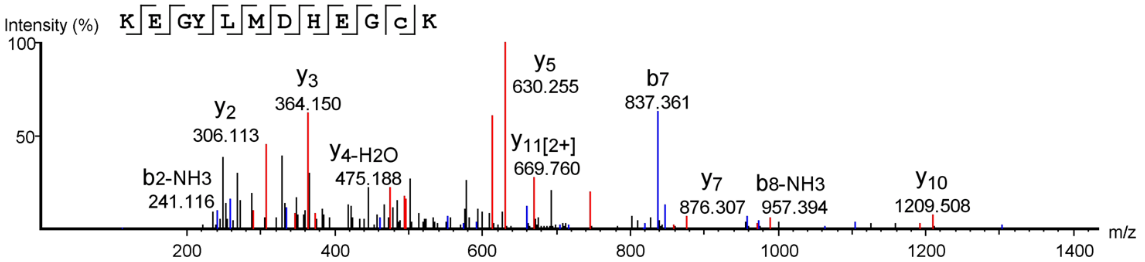

#	Immonium	b	b-H2O	b-NH3	a	c-H	b (2+)	Seq	y	y-H2O	y-NH3	y (2+)	#
1	101.108	129.103	111.092	112.150	101.108	145.122	65.051	K					12
2	102.055	258.137	240.135	241.116	230.150	274.165	129.573	E	1338.545	1320.535	1321.518	669.760	11
3	30.034	315.167	297.156	298.140	287.172	331.186	158.083	G	1209.508	1191.436	1192.439	605.251	10
4	136.076	478.230	460.199	461.203	450.235	494.249	239.615	Y	1152.481	1134.471	1135.454	576.741	9
5	86.097	591.326	573.234	574.287	563.319	607.333	296.157	L	989.400	971.432	972.356	495.177	8
6	104.053	722.355	704.353	705.328	694.360	738.374	361.677	M	876.307	858.360	859.307	438.667	7
7	88.040	837.361	819.363	820.310	809.387	853.401	419.191	D	745.286	727.283	728.266	373.168	6
8	110.072	974.392	956.430	957.394	946.446	990.460	487.720	H	630.255	612.243	613.241	315.633	5
9	102.055	1103.469	1085.473	1086.456	1075.488	1119.502	552.215	E	493.194	475.188	476.181	247.104	4
10	30.034	1160.505	1142.494	1143.478	1132.510	1176.524	580.752	G	364.150	346.132	347.138	182.582	3
11	133.043	1320.535	1302.486	1303.508	1292.540	1336.554	660.758	C(+57.02)	307.128	289.133	290.122	154.072	2
12	101.108							K	147.113	129.102	130.086	74.056	1

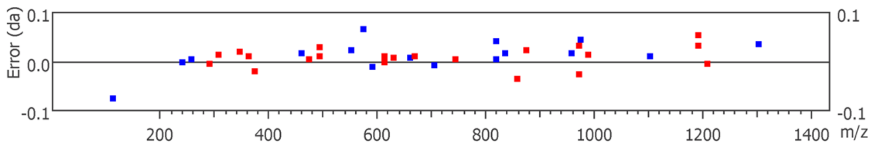

Figure S6. Spectrum from ion 1465.6329 tryptic peptides identified for Tb1 with ion table and error map.

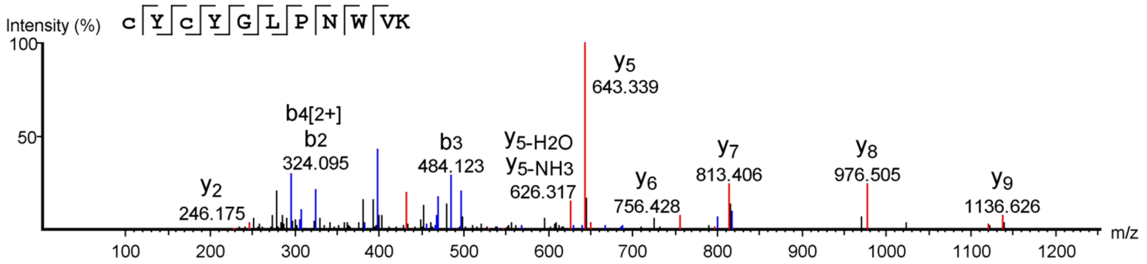

#	Immonium	b	b-H2O	b-NH3	a	c-H	b (2+)	Seq	y	y-H2O	y-NH3	y (2+)	#
1	133.043	161.038	143.028	144.011	133.043	177.058	81.019	C(+57.02)					11
2	136.076	324.095	306.088	307.075	296.101	340.121	162.551	Y	1299.619	1281.609	1282.592	650.291	10
3	133.043	484.123	466.204	467.091	456.200	500.152	242.566	C(+57.02)	1136.626	1118.545	1119.509	568.778	9
4	136.076	647.196	629.200	630.119	619.201	663.215	324.095	Y	976.505	958.514	959.498	488.763	8
5	30.034	704.217	686.292	687.190	676.222	720.236	352.609	G	813.406	795.451	796.377	407.231	7
6	86.097	817.304	799.261	800.254	789.306	833.320	409.151	L	756.428	738.430	739.413	378.720	6
7	70.066	914.354	896.344	897.327	886.359	930.373	457.677	P	643.339	625.333	626.317	322.178	5
8	87.056	1028.397	1010.386	1011.370	1000.402	1044.416	514.698	N	546.266	528.293	529.374	273.652	4
9	159.092	1214.476	1196.466	1197.449	1186.481	1230.495	607.738	W	432.256	414.250	415.234	216.630	3
10	72.081	1313.545	1295.534	1296.518	1285.550	1329.564	657.272	V	246.175	228.188	229.105	123.591	2
11	101.108							K	147.113	129.102	130.086	74.056	1

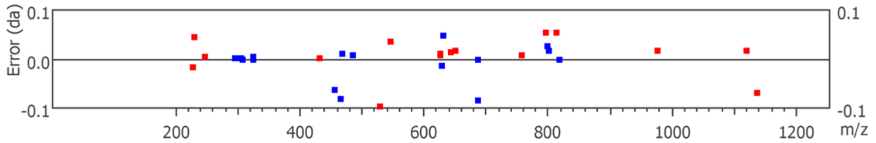

Figure S7. Spectrum from ion 1458.6425 tryptic peptides identified for Tb1 with ion table and error map.

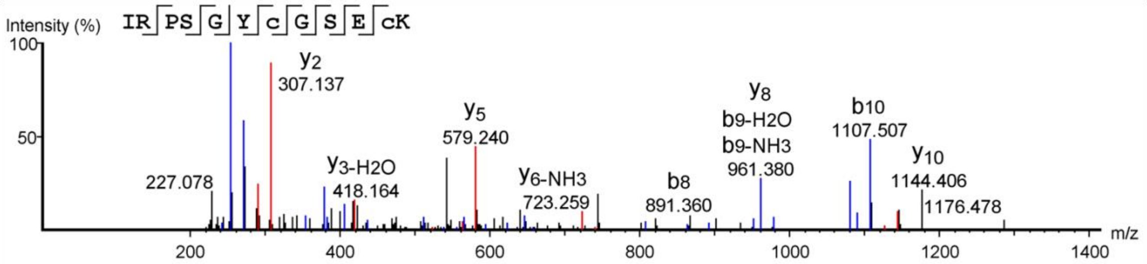

#	Immonium	b	b-H2O	b-NH3	a	c-H	b (2+)	Seq	y	y-H2O	y-NH3	y (2+)	#
1	86.097	114.092	96.081	97.065	86.097	130.111	57.546	I					12
2	129.114	270.186	252.182	253.160	242.123	286.212	135.596	R	1300.541	1282.530	1283.514	650.770	11
3	70.066	367.246	349.235	350.219	339.251	383.265	184.123	P	1144.406	1126.469	1127.413	572.720	10
4	60.045	454.278	436.202	437.251	426.283	470.297	227.639	S	1047.387	1029.376	1030.360	524.193	9
5	30.034	511.285	493.289	494.272	483.304	527.318	256.150	G	960.360	942.344	943.328	480.677	8
6	136.076	674.363	656.352	657.381	646.364	690.382	337.681	Y	903.333	885.323	886.307	452.167	7
7	133.043	834.393	816.383	817.366	806.423	850.412	417.697	C(+57.02)	740.298	722.260	723.259	370.635	6
8	30.034	891.360	873.404	874.388	863.430	907.434	446.207	G	580.250	562.185	563.213	290.620	5
9	60.045	978.409	960.360	961.380	950.401	994.466	489.723	S	523.235	505.207	506.191	262.109	4
10	102.055	1107.507	1089.410	1090.429	1079.509	1123.508	554.256	E	436.202	418.164	419.159	218.593	3
11	133.043	1267.520	1249.509	1250.493	1239.525	1283.539	634.260	C(+57.02)	307.137	289.125	290.116	154.072	2
12	101.108							K	147.113	129.102	130.086	74.056	1

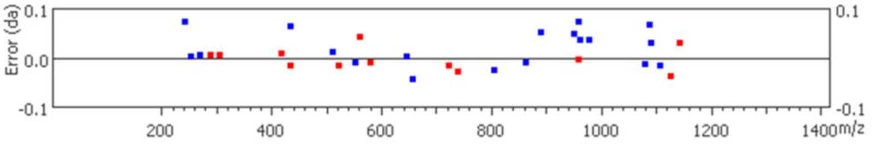

Figure S8. Spectrum from ion 1412.6177 tryptic peptides identified for Tb1 with ion table and error map.

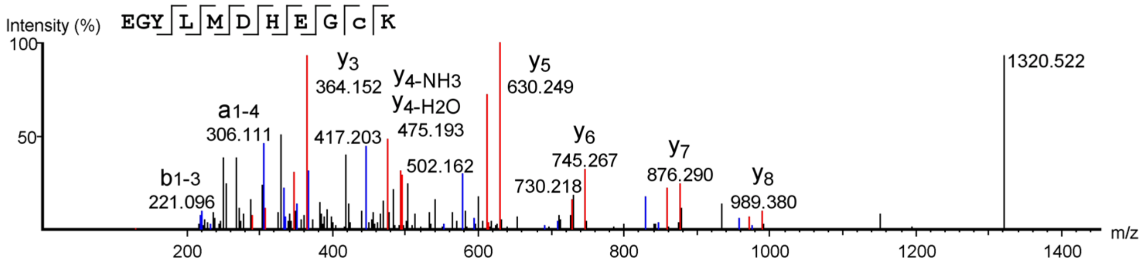

#	Immonium	b	b-H2O	b-NH3	a	c-H	b (2+)	Seq	y	y-H2O	y-NH3	y (2+)	#
1	102.055	130.050	112.040	113.023	102.055	146.070	65.525	E					11
2	30.034	187.072	169.061	170.045	159.077	203.091	94.036	G	1209.503	1191.492	1192.476	605.251	10
3	136.076	350.120	332.117	333.108	322.140	366.127	175.568	Y	1152.481	1134.471	1135.454	576.741	9
4	86.097	463.219	445.197	446.202	435.224	479.238	232.115	L	989.380	971.369	972.391	495.157	8
5	104.053	594.250	576.249	577.235	566.265	610.279	297.630	M	876.290	858.289	859.316	438.667	7
6	88.040	709.281	691.264	692.260	681.292	725.306	355.143	D	745.267	727.285	728.247	373.147	6
7	110.072	846.306	828.307	829.319	818.351	862.365	423.673	H	630.249	612.243	613.244	315.633	5
8	102.055	975.342	957.299	958.302	947.393	991.407	488.194	E	493.198	475.193	476.191	247.104	4
9	30.034	1032.410	1014.399	1015.383	1004.415	1048.429	516.705	G	364.152	346.140	347.096	182.582	3
10	133.043	1192.440	1174.430	1175.413	1164.445	1208.459	596.720	C(+57.02)	307.129	289.133	290.109	154.072	2
11	101.108							K	147.113	129.057	130.086	74.056	1

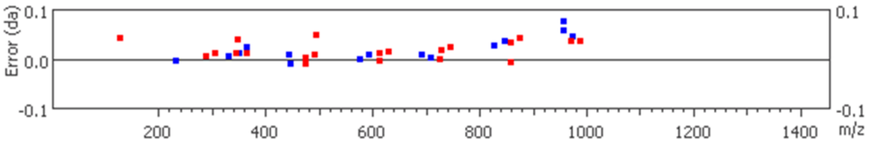

Figure S9. Spectrum from ion 1337.538 tryptic peptides identified for Tb1 with ion table and error map.

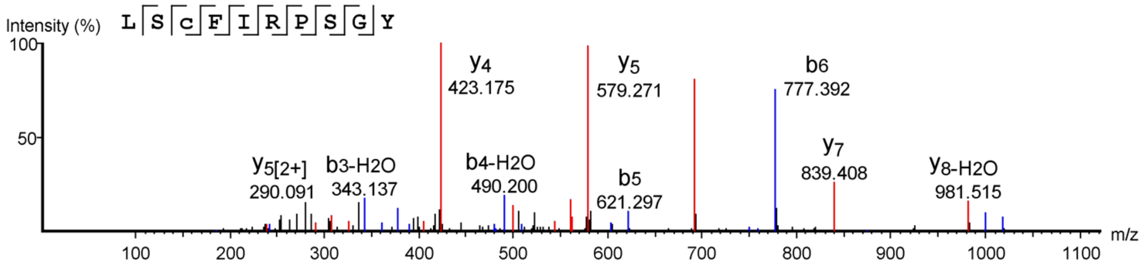

#	Immonium	b	b-H2O	b-NH3	a	c-H	b (2+)	Seq	y	y-H2O	y-NH3	y (2+)	#
1	86.097	114.092	96.081	97.065	86.097	130.111	57.546	L					10
2	60.045	201.124	183.113	184.097	173.129	217.143	101.062	S	1086.504	1068.493	1069.477	543.714	9
3	133.043	361.134	343.137	344.128	333.160	377.123	181.077	C(+57.02)	999.392	981.515	982.445	500.228	8
4	120.081	508.230	490.200	491.196	480.214	524.242	254.611	F	839.408	821.430	822.414	420.220	7
5	86.097	621.297	603.275	604.280	593.312	637.326	311.154	I	692.354	674.362	675.346	346.686	6
6	129.114	777.392	759.364	760.381	749.376	793.427	389.224	R	579.271	561.266	562.257	290.091	5
7	70.066	874.449	856.450	857.434	846.466	890.480	437.730	P	423.175	405.159	406.160	212.094	4
8	60.045	961.493	943.482	944.466	933.498	977.512	481.227	S	326.126	308.098	309.108	163.567	3
9	30.034	1018.527	1000.455	1001.487	990.519	1034.534	509.757	G	239.098	221.092	222.076	120.051	2
10	136.076							Y	182.081	164.071	165.054	91.541	1

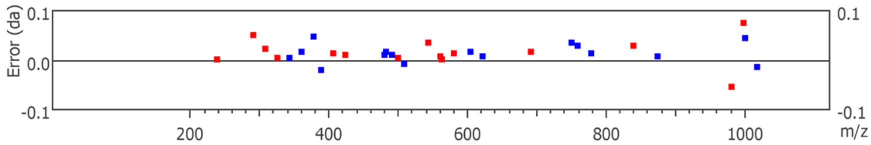

Figure S10. Spectrum from ion 1198.5804 tryptic peptides identified for Tb1 with ion table and error map.

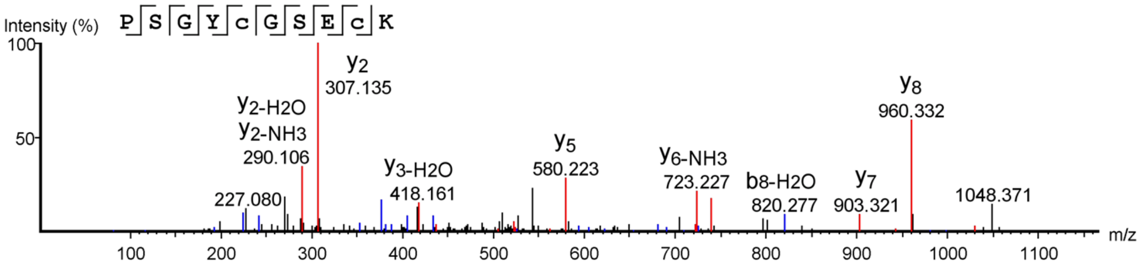

#	Immonium	b	b-H2O	b-NH3	a	c-H	b (2+)	Seq	y	y-H2O	y-NH3	y (2+)	#
1	70.066	98.061	80.050	80.974	70.066	114.080	49.530	P					10
2	60.045	185.093	167.082	168.066	157.098	201.112	93.046	S	1047.387	1029.329	1030.460	524.189	9
3	30.034	242.109	224.098	225.099	214.119	258.133	121.557	G	960.332	942.389	943.342	480.677	8
4	136.076	405.160	387.140	388.144	377.170	421.197	203.089	Y	903.321	885.323	886.307	452.167	7
5	133.043	565.208	547.197	548.181	537.213	581.227	283.104	C(+57.02)	740.251	722.243	723.227	370.635	6
6	30.034	622.200	604.198	605.203	594.154	638.249	311.615	G	580.223	562.178	563.213	290.620	5
7	60.045	709.228	691.228	692.235	681.168	725.192	355.131	S	523.201	505.206	506.191	262.109	4
8	102.055	838.304	820.277	821.277	810.309	854.323	419.652	E	436.165	418.161	419.159	218.593	3
9	133.043	998.285	980.276	981.308	970.340	1014.354	499.667	C(+57.02)	307.135	289.126	290.106	154.072	2
10	101.108							K	147.113	129.102	130.086	74.056	1

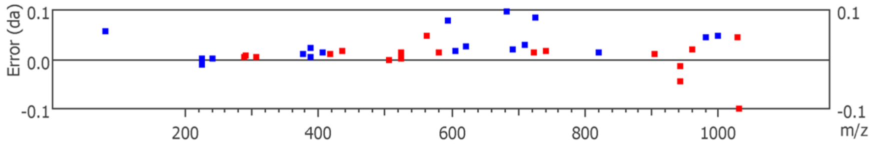

Figure S11. Spectrum from ion 1143.4325 tryptic peptides identified for Tb1 with ion table and error map.

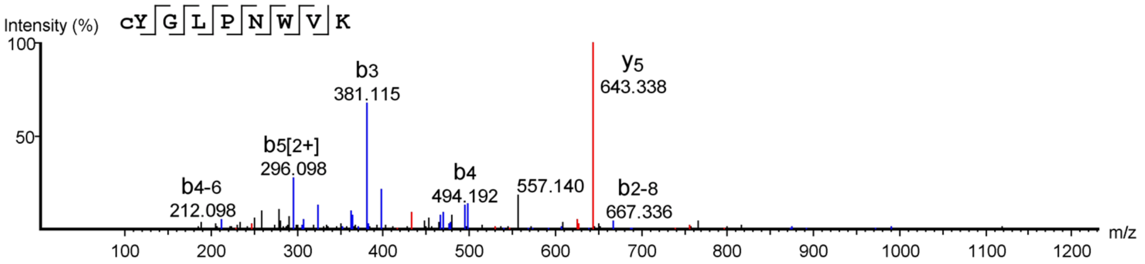

#	Immonium	b	b-H2O	b-NH3	a	c-H	b (2+)	Seq	y	y-H2O	y-NH3	y (2+)	#
1	133.043	161.038	143.028	144.011	133.043	177.058	81.019	C(+57.02)					9
2	136.076	324.092	306.080	307.070	296.098	340.121	162.551	Y	976.525	958.514	959.498	488.763	8
3	30.034	381.115	363.110	364.105	353.150	397.142	191.062	G	813.462	795.501	796.402	407.231	7
4	86.097	494.192	476.186	477.166	466.200	510.227	247.604	L	756.421	738.378	739.451	378.720	6
5	70.066	591.304	573.193	574.233	563.265	607.341	296.098	P	643.338	625.331	626.334	322.178	5
6	87.056	705.303	687.255	688.311	677.308	721.322	353.150	N	546.275	528.293	529.270	273.652	4
7	159.092	891.338	873.344	874.337	863.387	907.401	446.191	W	432.248	414.250	415.223	216.630	3
8	72.081	990.397	972.481	973.424	962.456	1006.470	495.725	V	246.176	228.171	229.131	123.591	2
9	101.108							K	147.113	129.102	130.086	74.056	1

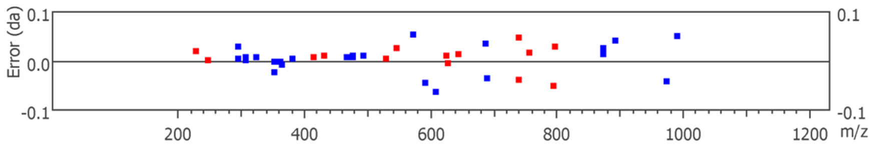

Figure S12. Spectrum from ion 1135.5485 tryptic peptides identified for Tb1 with ion table and error map.

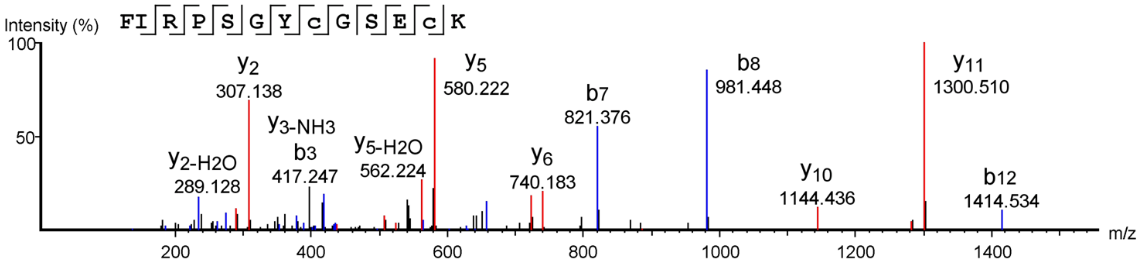

#	Immonium	b	b-H2O	b-NH3	a	c-H	b (2+)	Seq	y	y-H2O	y-NH3	y (2+)	#
1	120.081	148.076	130.066	131.049	120.081	164.095	74.538	F					13
2	86.097	261.155	243.150	244.133	233.161	277.179	131.080	I	1413.625	1395.614	1396.598	707.312	12
3	129.114	417.247	399.251	400.150	389.235	433.183	209.131	R	1300.510	1282.623	1283.514	650.770	11
4	70.066	514.314	496.304	497.212	486.319	530.333	257.657	P	1144.436	1126.429	1127.413	572.720	10
5	60.045	601.345	583.336	584.319	573.351	617.365	301.173	S	1047.387	1029.376	1030.360	524.200	9
6	30.034	658.354	640.375	641.341	630.319	674.387	329.684	G	960.355	942.344	943.328	480.677	8
7	136.084	821.376	803.420	804.404	793.436	837.450	411.215	Y	903.333	885.382	886.307	452.167	7
8	133.043	981.448	963.451	964.435	953.467	997.481	491.231	C(+57.02)	740.183	722.195	723.232	370.635	6
9	30.034	1038.483	1020.472	1021.456	1010.488	1054.502	519.742	G	580.222	562.224	563.238	290.620	5
10	60.045	1125.515	1107.505	1108.488	1097.520	1141.534	563.238	S	523.195	505.207	506.191	262.109	4
11	102.055	1254.558	1236.547	1237.531	1226.563	1270.577	627.812	E	436.162	418.175	419.148	218.593	3
12	133.043	1414.534	1396.578	1397.561	1386.593	1430.608	707.794	C(+57.02)	307.138	289.128	290.116	154.072	2
13	101.108							K	147.113	129.102	130.086	74.056	1

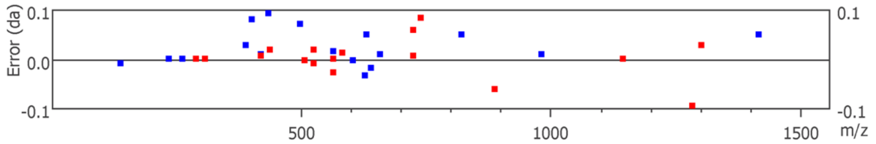

Figure S13. Spectrum from ion 1559.686 tryptic peptides identified for Tb1 with ion table and error map.

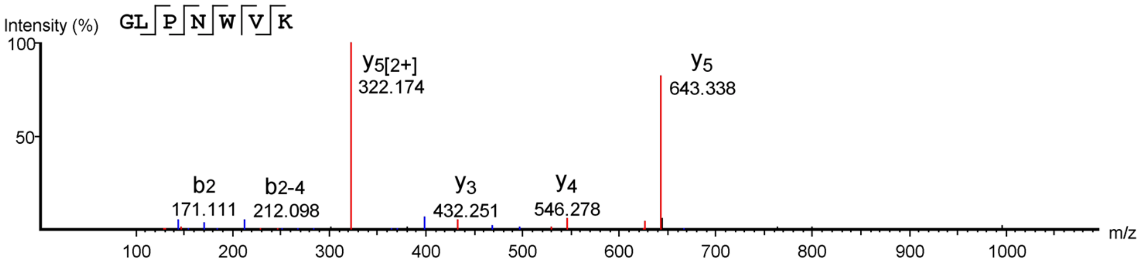

#	Immonium	b	b-H2O	b-NH3	a	c-H	b (2+)	Seq	y	y-H2O	y-NH3	y (2+)	#
1	30.034	58.029	40.019	41.002	30.034	74.048	29.515	G					7
2	86.097	171.111	153.103	154.066	143.115	187.133	86.057	L	756.440	738.430	739.413	378.720	6
3	70.066	268.139	250.156	251.099	240.171	284.104	134.583	P	643.338	625.306	626.311	322.174	5
4	87.056	382.209	364.139	365.182	354.214	398.175	191.605	N	546.278	528.293	529.236	273.652	4
5	159.092	568.288	550.278	551.261	540.293	584.307	284.644	W	432.251	414.250	415.234	216.630	3
6	72.081	667.387	649.346	650.330	639.362	683.376	334.178	V	246.181	228.151	229.154	123.591	2
7	101.108							K	147.109	129.100	129.995	74.056	1

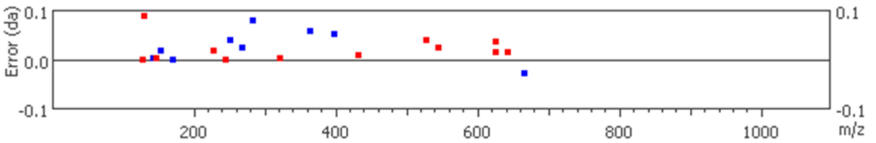

Figure S14. Spectrum from ion 812.4545 tryptic peptides identified for Tb1 with ion table and error map.

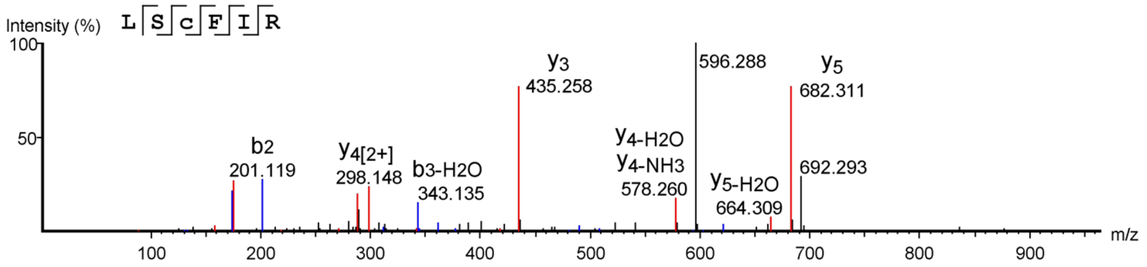

#	Immonium	b	b-H2O	b-NH3	a	c-H	b (2+)	Seq	y	y-H2O	y-NH3	y (2+)	#
1	86.097	114.092	96.081	97.065	86.097	130.071	57.546	L					6
2	60.045	201.119	183.113	184.097	173.125	217.143	101.062	S	682.311	664.309	665.307	341.665	5
3	133.040	361.151	343.135	344.137	333.160	377.104	181.077	C(+57.02)	595.302	577.242	578.260	298.148	4
4	120.081	508.213	490.199	491.196	480.239	524.242	254.611	F	435.258	417.197	418.244	218.067	3
5	86.097	621.295	603.258	604.280	593.248	637.326	311.218	I	288.197	270.186	271.176	144.602	2
6	129.114							R	175.116	157.108	158.090	88.038	1

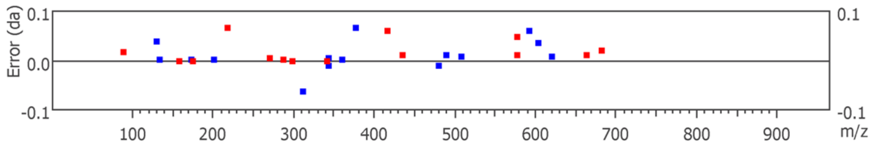

Figure S15. Spectrum from ion 794.4109 tryptic peptides identified for Tb1 with ion table and error map.

Table S1. List of peptides identified for Tb1.

Peptide	-10lgP	Mass	ppm	m/z	RT	Scan
C(+57.02*)AWPAC(+57.02*)YC(+57.02*)YGLPNWVK	49.71	2043.8794	41.8	10.229.897	0.49	270
LSC(+57.02*)FIRPSGYC(+57.02*)GSEC(+57.02*)K	62.56	1919.8328	-25.8	6.409.350	0.31	149
KGSSGYC(+57.02*)AWPAC(+57.02*)YC(+57.02*)Y	51	1828.7007	-18.6	9.153.406	0.38	258
FIRPSGYC(+57.02*)GSEC(+57.02*)K	45.78	1559.686	-18.5	5.208.930	0.25	67
KGSSGYC(+57.02*)AWPAC(+57.02*)Y	44.06	1505.6067	-35.1	7.537.842	0.34	216
KEGYLMDHEGC(+57.02*)K	46.59	1465.6329	-124.4	7.337.326	0.21	210
C(+57.02*)YC(+57.02*)YGLPNWVK	44.38	1458.6425	-26.2	7.303.094	0.42	208
IRPSGYC(+57.02*)GSEC(+57.02*)K	31.02	1412.6177	5.5	7.073.200	0.2	197
EGYLMDHEGC(+57.02*)K	53.48	1337.538	-23.4	6.697.606	0.24	177
LSC(+57.02*)FIRPSGY	44.89	1198.5804	-22.3	6.002.841	0.35	127
PSGYC(+57.02*)GSEC(+57.02*)K	48.93	1143.4325	-23.5	5.727.101	0.17	98
C(+57.02*)YGLPNWVK	37.23	1135.5485	-33.5	5.687.625	0.4	90
GLPNWVK	27.14	812.4545	-25.6	4.072.241	0.32	18
LSC(+57.02*)FIR	28.06	794.4109	-20.9	3.982.044	0.27	12

* +57.02: Carbamidomethylation.
